# Investigation of Urinary Exosome Metabolic Patterns in Membranous Nephropathy by Titania‐Assisted Intact Exosome Mass Spectrometry

**DOI:** 10.1002/smsc.202100118

**Published:** 2022-02-09

**Authors:** Haolin Chen, Ning Zhang, Yonglei Wu, Chenjie Yang, Qionghong Xie, Chunhui Deng, Nianrong Sun

**Affiliations:** ^1^ Department of Chemistry and Institutes of Biomedical Sciences Fudan University Shanghai 200433 P. R. China; ^2^ Division of Nephrology Huashan Hospital Fudan University Shanghai 200040 P. R. China; ^3^ Department of Gastroenterology and Hepatology Zhongshan Hospital Fudan University Shanghai 200032 China

**Keywords:** diagnosis, exosomes, mass spectrometry, membranous nephropathy, metabolites, titania

## Abstract

Exosomes are regarded as the emerging potential targets for liquid biopsy and bioprocess study owing to their abundant inclusive cargos that carry significant disease information. In addition, metabolites have been promising biomarkers for diagnosis. However, little metabolic research on exosomes is carried out by now. Herein, the mix‐crystal titania‐assisted laser desorption/ionization mass spectrometry (LDI‐MS) method is established, which features fast speed, high throughput, and efficiency, to directly extract urinary exosome metabolic patterns of healthy controls (HC) and membrane nephropathy (MN) patients. Besides, this method is also adopted to acquire the primitive urinary metabolic patterns from the same samples for comparison. By taking advantage of principal component analysis, unpaired parametric *t*‐test, and orthogonal partial least squares discriminant analysis on the exosome metabolic patterns, 27 significant *m/z* signals are filtrated, which possess more prominent differentiation capacity toward HC and MN patients (AUC = 0.942), and hold greater potential in MN diagnosis, compared to primitive urine (AUC = 0.801). The work reveals the important clinical value of exosome metabolic analysis, and paves a way to exosome‐based diagnosis at metabolomic level toward large‐scale clinical use.

## Graphical abstract

Herein, the urinary exosome metabolic patterns from healthy controls (HC) and membrane nephropathy (MN) patients are obtained through mix‐crystal titania‐assisted laser desorption/ionization mass spectrometry (LDI‐MS) with superior performance. Utilizing statistical analysis, 27 significant *m/z* signals are picked out, which more significantly differentiate MN patients from HC, and hold greater potential in MN diagnosis, compared to the urinary metabolic patterns themselves.

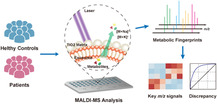

## 1 Introduction

Exosomes are small extracellular vesicles (EVs) with phospholipid bilayer, the diameter of which is about 50–150 nm. They can be secreted by nearly all types of cells through the fusion of multivesicular bodies (MVBs) with plasma membrane,^[^ ^1^ ^]^ and extensively exist in body fluids like blood,^[^ ^2^ ^]^ urine,^[^ ^3^ ^]^ tear,^[^ ^4^ ^]^ and breast milk.^[^ ^5^ ^]^ Moreover, a trove of cellular cargos, including transmembrane and cytoplasmic proteins, nucleic acids, lipids, and metabolites, are contained in exosomes. These cargos endow exosomes with strong ability to reflect the physiological and pathological states of parental cells, and play an irreplaceable role in intercellular communication,^[^ ^6^ ^]^ immunomodulation,^[^ ^7^ ^]^ and the occurrence and development of diseases.^[^ ^8^ ^]^ Therefore, exosomes have been regarded to be promising in screening biomarkers and performing liquid biopsies.

Compared to nucleic acids and proteins, it has been known that metabolites are more likely to reflect the real‐time dynamic changes of biological systems because they are intermediates or final products of biochemical reactions. Besides, because of the signal amplification effect of the regulation based on genetic and proteinic levels and relatively fewer types, metabolites are more favorable as biomarkers for disease diagnosis.^[^ ^9^ ^]^ Although metabolomics has been a rapidly developing technique for identification and quantitation of small molecular metabolites (<1000 Da) in organism at a specific time and under a particular environment,^[^ ^10^ ^]^ little metabolomics research on exosomes was carried out by now. As a consequence, it is quite necessary to develop advanced technology for investigating the metabolic patterns of exosomes.

Matrix‐assisted laser desorption/ionization mass spectrometry (LDI‐MS) with the ability to analyze samples of microliter volume in a few seconds has great potential in large‐scale clinical use.^[^ ^11^ ^]^ Notably, the performance of LDI‐MS is normally determined by the property of matrix. The traditional organic matrices such as α‐cyano‐4‐hydroxy cinnamic acid (CHCA) and 2,5‐dihydroxy‐benzoic acid (DHB) will be dissociated and form severe background interference in the low molecular weight region (<800 Da) during LDI process, limiting the application of LDI‐MS in metabolite detection.^[^ ^12^ ^]^ Furthermore, the cocrystallization heterogeneity of these conventional matrices and analytes results in poor reproducibility and brings difficulty to the quantitation of analytes by LDI‐MS.^[^ ^13^ ^]^ Surprisingly, given excellent light absorption properties, homogeneous dispersibility, large surface area, and multiple chemical surface modification, many nanomaterials have been selected as matrices of LDI‐MS to overcome the above shortcomings.^[^ 14, 15, 16, 17 ^]^ Especially, among the developed matrix nanomaterials, titania can effectively absorb UV light from laser source of LDI‐MS to exert photocatalytic properties, thereby facilitating the desorption/ionization of analytes.^[^ 18, 19 ^]^ In this regard, we speculate titania will be a desirable matrix for revealing exosome metabolic patterns by LDI‐MS.

Membranous nephropathy (MN) is a common pathogeny of nephropathy syndrome in adults.^[^ 20, 21 ^]^ Patients with MN type of nephropathy syndrome confront severe morbidity and mortality because of thromboembolic and cardiovascular complications.^[^ ^22^ ^]^ In clinic, renal biopsy is the gold standard for MN diagnosis, yet this traumatic examination makes a high claim for the operation and patient. Moreover, as the auxiliary mean of MN detection, serum antibody assay is process‐complexing, time‐consuming and costly.^[^ 23, 24 ^]^ As well known, urine is a biofluid that closely relates to the pathological states of kidney, and possesses the advantages of large volume, affluent exosome content, and noninvasive collection. Hence, urinary exosome metabolic analysis based on LDI‐MS for MN diagnosis is strongly anticipated. Herein, we selected a designer mix‐crystal titania to assist LDI‐MS to obtain the urinary exosome metabolic patterns for MN diagnosis. Owing to the excellent electron transferability, the mix‐crystal titania owned superior reproducibility, sensitivity, and selectivity for effective extraction of metabolic patterns. Combining the resultant exosome metabolic patterns with statistical analysis, the key signals that could significantly differentiate patients from healthy controls were filtrated. Notably, exosome metabolic patterns hold greater potential for MN diagnosis than primitive urine metabolic patterns. Our work sheds light on the exosome metabolomic research for monitoring and diagnosis of various diseases in large‐scale clinical use.

## 2 Results and Discussion

### 2.1 The Performance of Designer Titania‐Assisted LDI‐MS in Metabolite Detection

Owing to high UV absorption and photocatalyst activity, titania nanomaterials have been widely employed as the favorable matrix of LDI‐MS in metabolite detection. Titania can rapidly and efficiently absorb the energy from LDI‐MS laser, producing electron–hole pairs on the surface, and then metabolites are ionized through hole trapping. Moreover, the high photocatalyst activity of titania can effectively destroy the membrane structure during LDI process.^[^ ^25^ ^]^ P25 titania is the mixture of rutile and anatase crystalline phases with 3:1 ratio (21 nm in diameter on average), which has been extensively used in the heterogeneous photocatalytic redox process.^[^ 26, 27 ^]^ Significantly, mix‐crystal P25 titania has conspicuous UV absorption and photocatalyst activity than single‐crystal ones because the generation of transition points and catalytic “hot spots” between two phases allow rapid electron transfer, prominently decreasing its band gap.^[^ 28, 29 ^]^ Therefore, P25 titania is regarded as a better choice of acting as designer matrix materials of LDI‐MS. In this work, we plan to apply this designer titania matrix to assist LDI‐MS for direct extraction of exosomal metabolic patterns, which, as far as we know, has not been reported.

**Figure 1:**
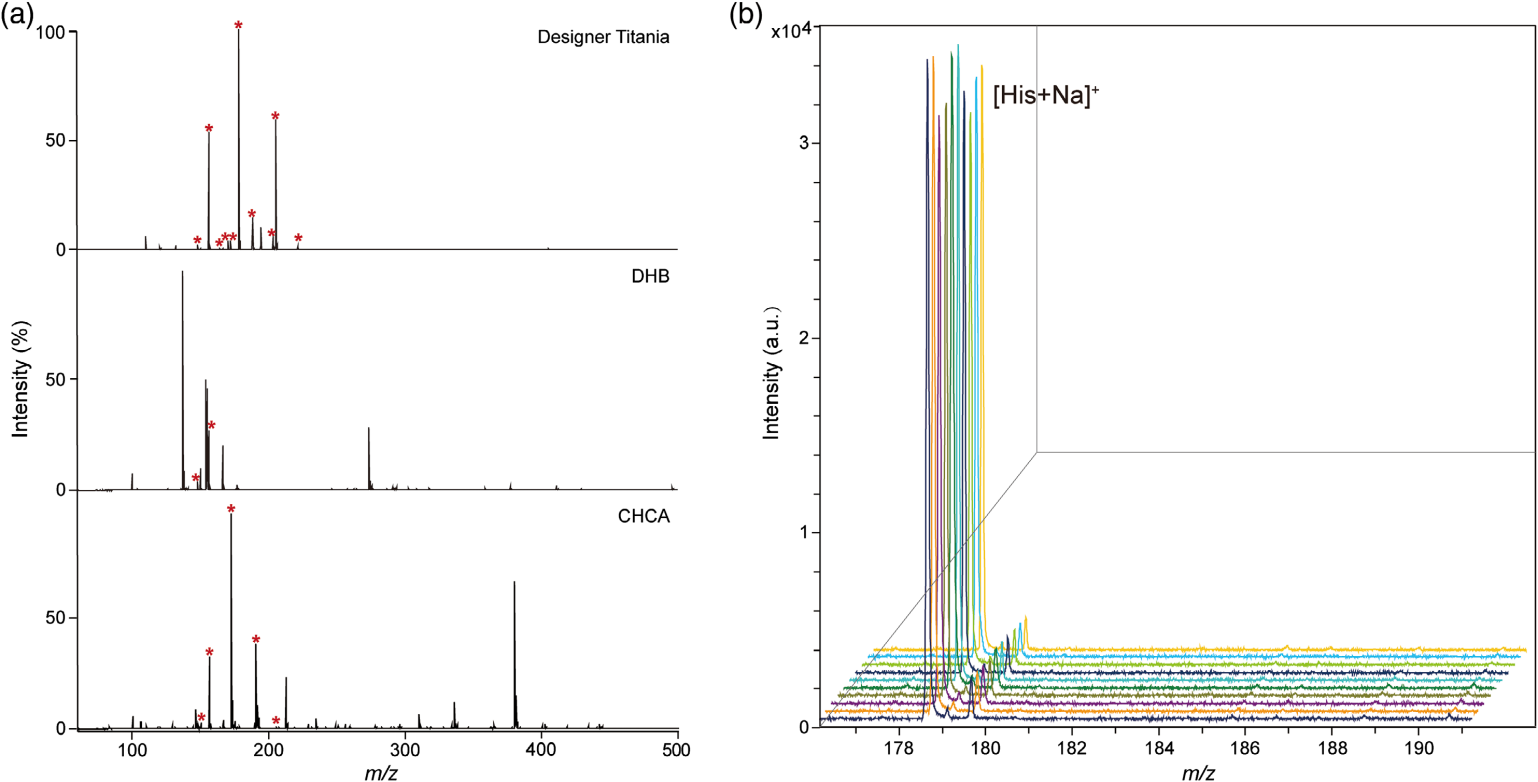
The performance of designer titania‐assisted LDI‐MS in metabolite detection. a) Mass spectra of 2 mg mL^−1^ standard metabolite mixture (glutamine acid, methionine, histidine, phenylalanine, taurine, aspartic acid, mannitol, and glucose) using designer titania, DHB, and CHCA as matrix, peaks with signal‐to‐noise ratio > 3 and relative intensity > 2% from the standard metabolites were annotated with asterisk. b) Mass spectra of histidine (200 μmol L^−1^) repeatedly acquired from one sample spot 10 times using designer titania as matrix.

The assistance performance of the designer titania matrix for LDI‐MS‐based metabolite detection was first investigated. For comparison, two single‐crystal titania nanomaterials (rutile and anatase) and two traditional organic matrices (CHCA and DHB) were applied at the same time. Here, eight metabolites including glutamine acid, methionine, histidine, phenylalanine, taurine, mannitol, and glucose were mixed as the test sample. As shown in **Figure** **1**a and S1, Supporting Information, the number of standard metabolites in the spectrum of designer titania is obviously more than two single‐crystal titania and two traditional organic matrices because the designer titania can efficiently absorb the energy from laser and then transfer the energy to the metabolites for enhancing the desorption and ionization. Moreover, compared to designer titania, severe background interference can be observed when using DHB or CHCA as matrix. This was because the high melting and boiling points of the designer titania matrix make it hard to be dissociated and ionized, thereby eliminating background interference in metabolic analysis, while traditional organic matrices were opposite.

It has been known the poor crystallization property of traditional organic matrices is another troublesome problem during LDI‐MS analysis, apart from the background interference. Heterogeneous cocrystal of matrix and analyte generally causes the generation of “sweet spot,” leading to low reproducibility in shot‐to‐shot and spot‐to‐spot LDI‐MS measurement. Therefore, we characterized the crystallization state of the mixture of histidine and the designer titania, CHCA, or DHB and explored the reproducibility. As shown in Figure S2, Supporting Information, the cocrystal of CHCA–histidine and DHB–histidine were unevenly aggregated, while the designer titania–histidine cocrystal formed a uniform film on the target plate. In theory, the homogeneous cocrystallization of the designer titania and analyte would guarantee good LDI‐MS reproducibility. We further verified the reproducibility of designer titania‐assisted LDI‐MS through repeatedly acquiring the spectra of histidine, including one sample spot for ten shots and ten sample spots. As shown in Figure 1b and S3, Supporting Information, LDI‐MS spectra and signal intensities of [His+Na]^+^ are reproducible, and the relative standard deviations for shot‐to‐shot and spot‐to‐spot assays are 5.60% and 2.64%, respectively, indicating that there was no “sweet spot” generation on designer titania‐assisted LDI‐MS analysis. Namely, great reproducibility of designer titania‐assisted LDI‐MS is observed as expected.

Additionally, high contents of salts and proteins in biofluids are the main hindrance during detection of metabolites, thereby we also tested the salt tolerance and protein endurance of designer titania. As demonstrated in Figure S4, Supporting Information, the designer titania‐assisted LDI‐MS shows superior selectivity toward metabolites in the biomimetic fluid. All eight standard metabolites could be detected with the presence of high salt (0.5 mol L^−1^ NaCl) and protein (2.5 mg mL^−1^ bovine serum albumin [BSA]) concentration. The principle for direct detection of metabolites surrounded by salts and proteins is probably the selective LDI process by nanostructures. Specifically, the nanocrevice of nanomaterials can trap small molecules instead of large ones due to size‐exclusive effect and transfer laser energy selectively in the LDI process. Further, the sensitivity of the designer titania was certified. The acceptable linearity was first obtained between the signal intensity and the concentration of analytes, in the range of 1–200 pmol for phenylalanine, mannitol, and glucose, 10–500 pmol for glutamic acid, and 1–100 pmol for methionine and cholesterol (*R* ^2^ > 0.99, Figure S5, Supporting Information). Surprisingly, we discovered that signal of the above metabolites could be facilely detected in low concentrations, with stronger signal‐to‐noise ratio compared to DHB matrix (Figure S6, Supporting Information).

### 2.2 Extraction of Exosomal Metabolic Patterns by Designer Titania‐Assisted LDI‐MS

**Figure 2:**
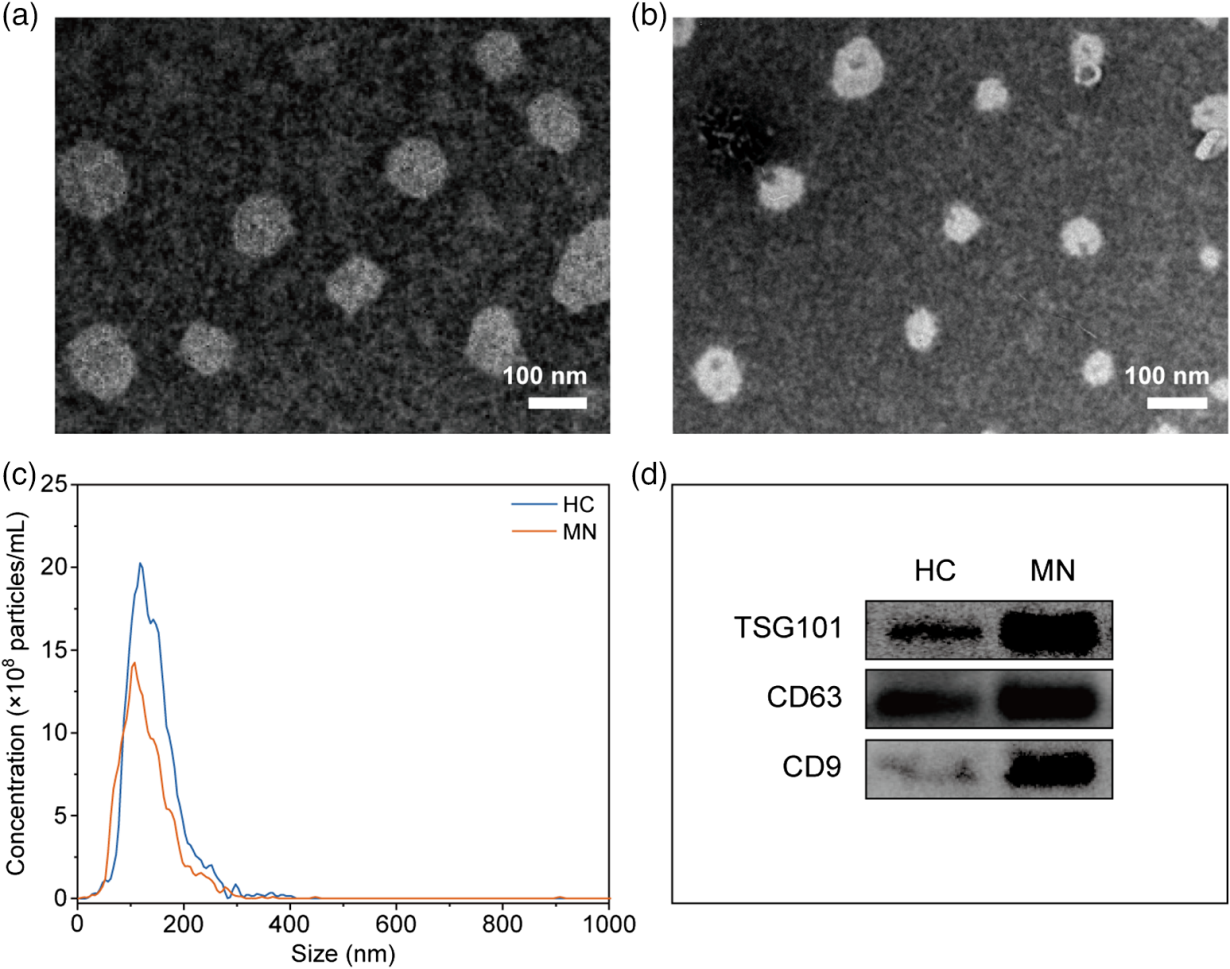
TEM images of isolated urinary exosomes from a) HC and b) MN patient. c) Size distribution of urinary exosomes measured by NTA. d) Western blot analysis against TSG101, CD63, and CD9 of urinary exosomes from HC and MN patient.

Encouraged by the above good performance including high reproducibility, sensitivity, and selectivity, designer titania‐assisted LDI‐MS was further applied to profile the metabolic patterns of urinary exosomes. In this work, we collected urine samples from ten HC and ten MN patients. Fisher's precision probability test (*p* > 0.9999) and Mann–Whitney *U* test (*p* = 0.7817) show there is no significant difference in gender and age distribution between HC and MN patients. The exosomes were isolated from these urine samples referring to our previous research.^[^ ^30^ ^]^ Transmission electron microscope (TEM) was first employed to characterize the morphology structure of the isolated urinary exosomes. From TEM images (**Figure** **2**a,b), these urinary exosomes are typical cup‐shaped structures, and their diameter distribution meets the definition of exosomes (about 50–120 nm). Then nanoparticle tracking analysis (NTA) was also adopted to measure the size distribution of the exosomes. As seen in Figure 2c, the obvious size peaks of exosomes from HC and MN patients are about 119 and 107 nm, respectively. Next, Western blot assay shows that urinary exosomes from both HC and MN patients were positive for TSG101, CD63, and CD9 markers (Figure 2d). The bands of these markers are particularly darker in MN patients than HC, preliminarily suggesting that exosome may possess the distinguishability and significance for MN detection.

### 2.3 Investigation of Urinary Exosome Metabolic Patterns in MN

**Scheme 1:**
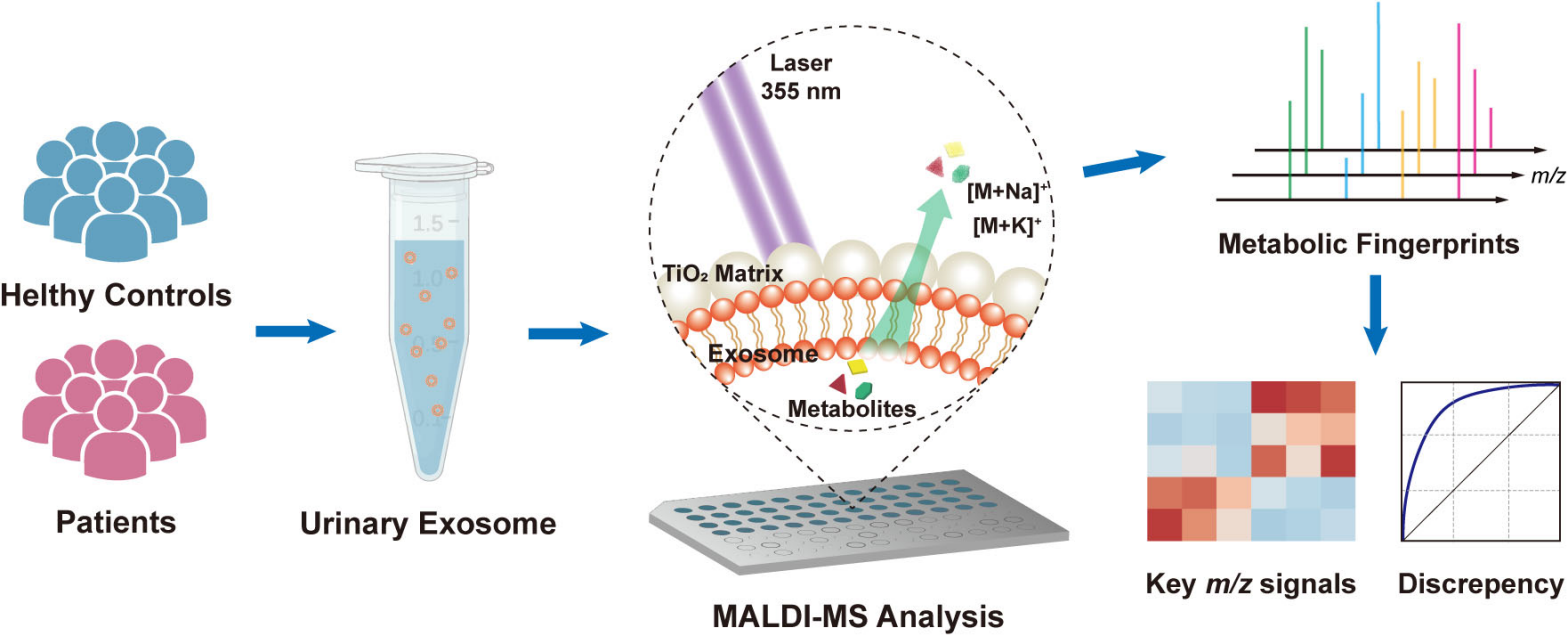
Workflow of titania‐assisted intact exosome mass spectrometry for investigating the metabolic patterns of urinary exosome.

**Figure 3:**
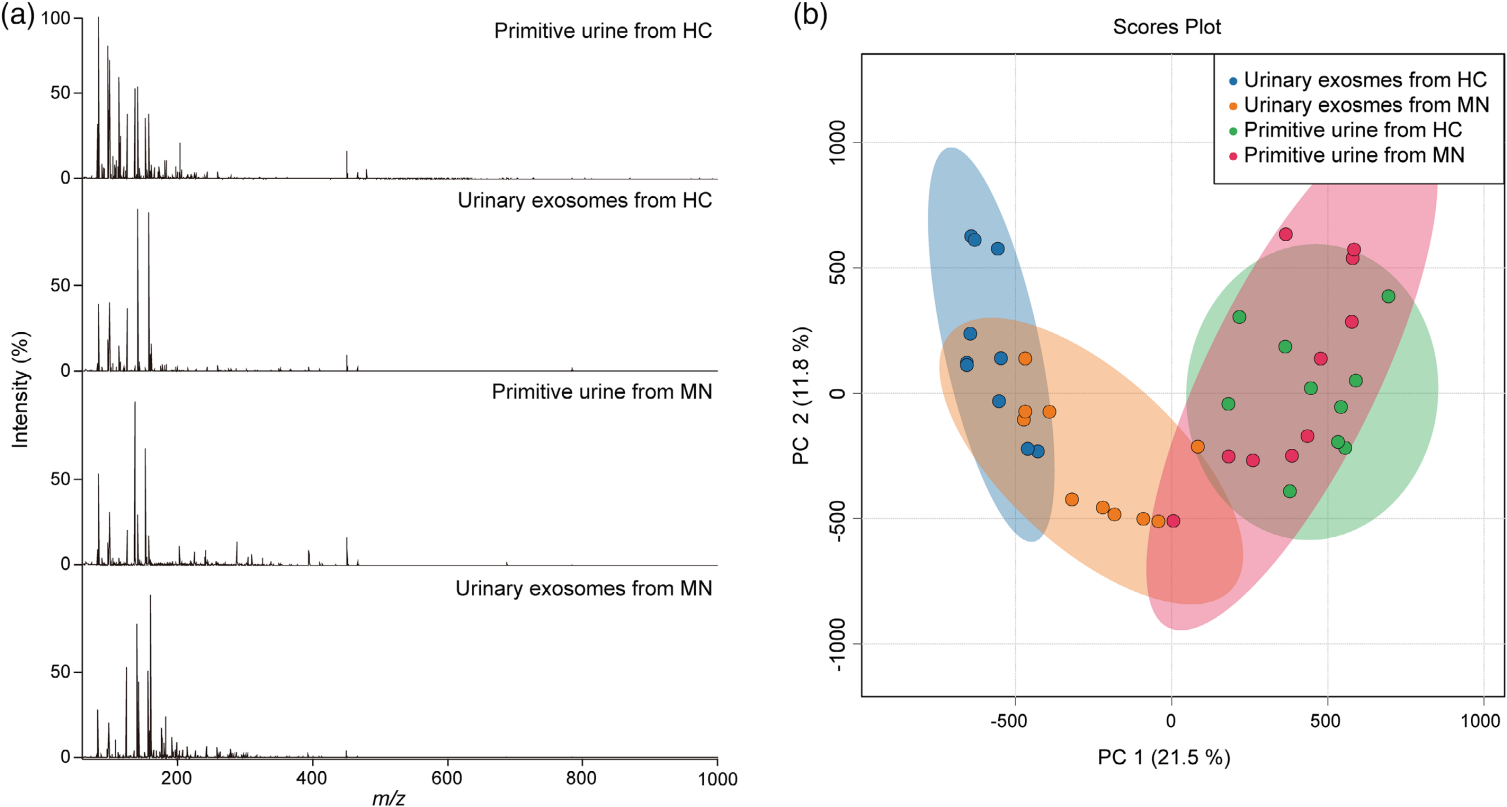
a) Typical metabolic patterns of the primitive urine samples and corresponding extracted exosomes from a HC and a MN patient. b) PCA scores plot based on metabolic patterns of the primitive urine samples and corresponding extracted exosomes.

We have preliminarily demonstrated the successful isolation of exosomes from urine samples, both healthy and of MN. Subsequently, the designer titania‐assisted LDI‐MS was employed to extract exosome metabolic patterns directly (**Scheme** **1**). For better illustration, we also directly acquired the metabolic patterns of primitive urine samples by designer titania‐assisted LDI‐MS. The typical MS spectra of the primitive urine samples and corresponding extracted exosomes from a HC and a MN patient are presented in **Figure** **3**a, wherein obvious difference can be observed. This result roughly confirms that we have efficiently obtained exosomal metabolic patterns by designer titania‐assisted intact exosome mass spectrometry, without any complicated metabolite extraction steps,^[^ 31, 32 ^]^ which is mainly attributed to the great contribution of promising matrix in LDI‐MS analysis. Briefly, after the designer titania matrix absorbs the energy of UV light, positive hole h+, hydroxyl radical ·OH, and hydrogen peroxide will be generated on its surfaces, helping destroy the membrane structure and release the inclusive metabolites. In the meantime, UV absorption and photocatalytic property of the designer titania matrix efficiently assist LDI‐MS metabolic detection.^[^ 25, 33 ^]^

After all the collected metabolic spectra were treated with peak extraction and peak alignment, we applied all *m/z* signals to statistical analysis. Principal component analysis (PCA) was first carried out based on metabolic patterns of the primitive urine samples and corresponding extracted exosomes, and the scores plot is shown in Figure 3b. The points corresponding to the primitive urine samples are distinctly separated from the urinary exosomes. Besides, separation can be also observed between urinary exosomes derived from HC and from MN to a certain extent, while no obvious difference can be observed between primitive HC urine and primitive MN urine. These results indicate that compared with primitive urine, urinary exosome metabolites distinguish MN from HC more strongly, and more closely link with the disease, thereby being more promising for diagnostics.

**Figure 4:**
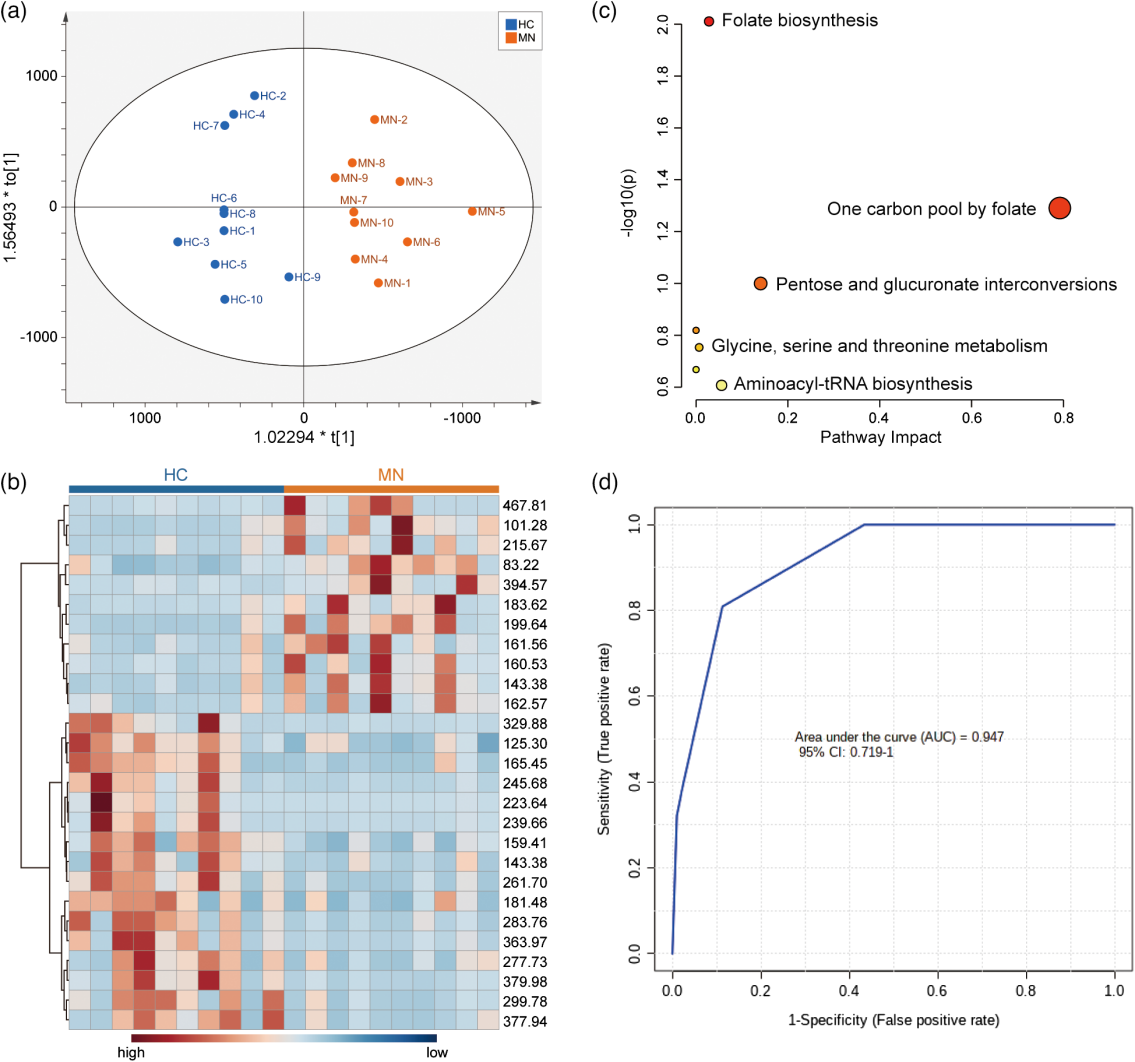
a) OPLS‐DA scores plot based on metabolic patterns of urinary exosomes. b) Heat map based on selected 27 significant *m/z* signals (VIP scores >1, *p* < 0.05, and fold change > 2). c) The pathway analysis of MN‐related metabolites. d) ROC curve based on 27 significant *m/z* signals.

To deeply explore the discrepancy of these exosome metabolic patterns between MN patients and HC, orthogonal partial least squares discriminant analysis (OPLS‐DA) was executed based on 322 *m/z* signals from urinary exosomes. The scores plot of OPLS‐DA showed clear group separation of HC and MN, with *R* ^2^ *Y*(cum) of 0.837 and *Q* ^2^(cum) of 0.504, displaying remarkable interpretability and predictability of the model (**Figure** **4**a). The permutation test using 100 iterations was proceeded, as the permutations plot shown in Figure S7, Supporting Information. All *R* ^2^ and *Q* ^2^ points on the right (analog values) are higher than those on the left (actual values), and the intercept of the regression line of *Q* ^2^ is less than 0.05, strongly illustrating that the model is not overfitted. Among the 322 *m/z* signals, a total of 46 *m/z* signals, with variable importance for the projection (VIP) scores greater than 1, were regarded as the significant contributors to the separation in the OPLS‐DA model (Figure S8, Supporting Information). In addition, unpaired parametric *t*‐test was performed, 72 *m/z* signals with *p* values less than 0.05 and fold change greater than 2 were also considered to possess statistical significance for discrimination of HC and MN groups (Figure S9, Supporting Information). Given the VIP values, *p* values, and fold change values, we ultimately selected 27 *m/z* signals as key features from the metabolic patterns of urinary exosomes (VIP scores > 1, *p* < 0.05, and fold change > 2, Table S1, Supporting Information). The heatmap of key *m/z* signals validates the visible discrepancy between MN patients and HC (Figure 4b). Multivariate receiver operating characteristic (ROC) analysis based on the key 27 *m/z* signals provided a desirable result, with the area under ROC curve (AUC) of 0.942 (Figure 4d). For comparison, the primitive urine metabolic patterns were also acquired, and the corresponding *m/z* signals meeting one of the above screening criteria (VIP scores > 1, or *p* < 0.05, and fold change > 2) are listed in Table S2, Supporting Information. It can be seen that 13 *m/z* signals presented VIP scores > 1, whereas only 4 *m/z* signals showed *p* < 0.05 and fold change > 2. There were only 2 *m/z* signals (*m/z* = 83.23 and *m/z* = 97.19) that met all the above screening criteria; as a result, ROC analysis showed relatively poorer discrimination performance (AUC = 0.801, Figure S10, Supporting Information) than aforementioned exosome metabolite signals (AUC = 0.942). Furthermore, 3 HC and 3 MN urinary exosomal samples were chosen as prediction set, while the remaining 14 samples as observations. Based on the 27 key *m/z* signals, OPLS‐DA model showed the prediction set could be accurately classified with AUC up to 1 (Figure S11, Supporting Information). These outcomes suggest that compared to primitive urine, the 27 key *m/z* signals from exosomal metabolic patterns possess the preferable discriminate ability for MN and HC, and have the potential for MN diagnosis in clinical use.

To verify the reliability of the key *m/z* signals and establish the further connection with MN, we conducted the HMDB database searching and mapping. Eighteen key *m/z* signals were assigned to corresponding metabolites (Table S1, Supporting Information). Thereinto, the *m/z* = 467.81 upregulated in MN is concluded as tetrahydrofolic acid, the upstream metabolite of methylene tetrahydrofolate reductase (MTHFR). MTHFR gene variation is the major cause of diabetic nephropathy;^[^ ^34^ ^]^ the overexpression of tetrahydrofolic acid reflects the aberrant folate metabolism and probably causes the emergence of MN. The *m/z* = 379.98 may belong to lactosamine, the precursor of *N*‐acetylgalactosamine. Abnormal *O*‐glycosylation of the IgA1 hinge region involves the pathogenesis of IgA nephropathy,^[^ ^35^ ^]^ and the decrease of lactosamine in patients may participate in this procedure, finally leading to the progression of MN. The pathway analysis of MN‐related metabolites also manifests five impactive pathways, including folate biosynthesis;^[^ ^36^ ^]^ one carbon pool by folate;^[^ ^37^ ^]^ pentose and glucuronate interconversions;^[^ ^38^ ^]^ glycine, serine, and threonine metabolism;^[^ ^39^ ^]^ and aminoacyl‐tRNA biosynthesis,^[^ ^40^ ^]^ which agrees with reported related researches (Figure 4c).

## 3 Conclusion

In summary, based on superior reproducibility, sensitivity, and selectivity of the mix‐crystal titania‐assisted LDI‐MS, we profiled the metabolic patterns of urinary exosomes with fast speed, high throughput, and efficiency. According to the exosomal metabolic patterns, the key mass signals were discovered, which can differentiate MN patients from healthy controls, exhibiting the better diagnostic potential for MN compared to primitive urinary metabolic patterns. Considering the easy obtainment of titania nanomaterials, time efficiency, and sample requirement at microliter level, the mix‐crystal titania‐assisted intact exosome MS promises large‐scale clinical application. Overall, this platform inspires a brand new solution of exosomal metabolic analysis toward precision medicine, and anticipates the diagnostic utility against various diseases.

## 4 Experimental Section

4.1

4.1.1

##### Harvest of Urine Samples

All urine samples (of ten healthy controls and ten MN patients) were collected from Shanghai Huashan Hospital and permission was obtained from the Ethics Committee of Huashan Hospital at Fudan University (394). All urine donators consented this research. Details of healthy donors and patients were described in Table S3, Supporting Information. First, morning midstream urine (≈200 mL) was collected in tubes, placed in ice for 2 h to precipitate impurities. Afterward, the urine samples were centrifuged at 2000 × *g* for 10 min, filtered by 0.22 μm filter to remove cells, dead cells, cell debris, and apoptotic blebs. At last, the filter liquor was concentrated through an ultrafiltration tube (Merck Millipore, 100 kDa) and immediately frozen at −80 ºC for further use. Notably, for the control experiment, 5 μL primitive urine samples without any treatment were subpackaged and stored at −80 °C beforehand.

##### Isolation and Characterization of Urinary Exosomes

Exosomes were isolated from concentrated urine samples referring the previous research of our group.^[^ ^30^ ^]^ Transmission electron microscope, nanoparticle tracking analysis, and Western blot assay were utilized to characterize the morphology, particle size distribution, and related proteins of isolated exosomes, respectively. The characterization procedures are given in the Supporting Information.

##### Matrix‐Assisted LDI‐MS Analysis

For the matrix preparation, P25 titania, anatase titania, and rutile titania were dispersed in water at a concentration of 1 mg mL^−1^, CHCA was prepared with a concentration of 5 mg mL^−1^ by dissolving in 0.1% TFA buffer (water/ACN, 50/50, v/v), and DHB was prepared with a concentration of 5 mg mL^−1^ by dissolving in 0.1% TFA buffer (water/ACN, 20/80, v/v). Then, 1 μL matrix solution was dropped onto the target plate (MTP 384 target plate ground steel BC), and it was dried at room temperature. Afterward, analytes (including standard solutions of metabolites, primitive urine samples, and corresponding isolated exosome samples) were added and dried on the spot of matrix. The mass spectra were obtained in reflector positive mode on an UlrafleXtreme MALDI‐TOF/TOF MS (Bruker Daltonic, Germany) equipped with a smartbeam Nd:YAG laser (355 nm) with the frequency of 2000 Hz and 70% intensity. The mass scan range was from 60 to 1000 *m/z*, and the device was calibrated by standard molecules beforehand. MALDI‐MS data were collected using Flexcontrol 3.4, each sample was tested with three replicates.

##### Statistical Analysis

Data detected by MALDI‐MS were extracted in Flexanalysis 3.4 software. Averaging mass spectra of the same samples and peak alignment were processed by MALDIquant^[^ ^41^ ^]^ package on R to build a matrix of *m/z* signals. The matrix was quantile‐normalized and pareto‐scaled, and then PCA and unpaired parametric *t*‐test were carried out using MetaboAnalyst 4.0 service^[^ ^42^ ^]^ (McGill University, Montreal, Canada). OPLS‐DA was performed using SIMCA‐P 14.1 software (MKS Umetrics, Umeå, Sweden). ROC curve construction and the AUC calculation were performed using MetaboAnalyst 4.0 service. Metabolite retrieval of the key *m/z* signals was processed on human metabolome database (HMDB) 4.0 (http://www.hmdb.ca/).^[^ ^43^ ^]^ The pathway analysis was implemented through MetaboAnalyst 4.0 service.

## Conflict of Interest

The authors declare no conflict of interest.

## Supporting information

Supplementary Material

## Data Availability

Data available on request from the authors.
