# Supplementary material for: Investigation of Urinary Exosome Metabolic Patterns in Membranous Nephropathy by Titania‐Assisted Intact Exosome Mass Spectrometry
Source: Small Sci. 2022 Feb 9;2(5):2100118. doi: 10.1002/smsc.202100118 (PMC11935978; doi:10.1002/smsc.202100118)
Supplement: Supplementary file 1 — Supplementary Material [file SMSC-2-2100118-s001.pdf]

Supporting Information

Investigation of urinary exosome metabolic patterns in membranous nephropathy by titania-assisted intact exosome mass spectrometry

Haolin Chen, Ning Zhang, Yonglei Wu, Chenjie Yang, Qionghong Xie, Chunhui Deng,* and Nianrong Sun **

Correspondence

*N. Sun, e-mail: sunnianrong@fudan.edu.cn

*C. Deng, e-mail: chdeng@fudan.edu.cn

*Q. Xie, e-mail: qionghongxie@fudan.edu.cn

Contents

Supplementary experimental procedures

Materials and chemicals, characterization of exosomes from HC and MN patients.

Supplementary figures

Figure S1. Mass spectra of 2 mg/mL standard metabolites mixture using anatase titania and rutile titania.

Figure S2. Images of DHB/CHCA/designer titania-histidine cocrystal dispersed on the target plate.

Figure S3. Mass signal intensities of histidine (200 μmol/L) repeatedly acquired from 10 sample spots using designer titania as matrix.

Figure S4. Mass spectra of 1 mg/mL standard metabolites mixture in 0.5 mol/L NaCl and 2.5 mg/mL BSA using designer titania as matrix.

Figure S5. The calibration curve of standard metabolites obtained by designer titania-assisted LDI-MS.

Figure S6. Mass spectra of 10 pmol phenylalanine, 50 pmol glutamic acid, 5 pmol methionine, 5 pmol mannitol, 5 pmol glucose and 50 pmol cholesterol using designer titania and DHB as matrix.

Figure S7. The Permutations Plot for OPLS-DA of HC vs MN patients.

Figure S8. Dotchart of 46 *m/z* signals with VIP score more than 1 between HC and MN patients.

Figure S9. Volcano plot based on metabolic patterns of urinary exosomes.

Figure S10. ROC curve based on urine metabolic patterns.

Figure S11. ROC curve of classification of prediction set.

Supplementary tables

Table S1. 27 significant *m/z* signals selected from metabolic patterns of urinary exosomes.

Table S2. The related *m/z* signals from metabolic patterns of primitive urine.

Table S3. Summary of 10 HC and 10 MN patients.

Supplementary experimental procedures

Materials and chemicals

Rutile titania and anatase titania were purchased from J&K Scientific (Beijing, China). P25 titania was obtained from Evonik Degussa (Essen, Germany). 2,5-Dihydroxybenzoic acid (DHB), α-Cyano-4-hydroxycinnamic acid (CHCA), D-Glutamic acid, D-Methionine, L-Histidine, L-Phenylalanine, Taurine, and L-Aspartic acid were obtained from Sigma-Aldrich (Darmstadt, Germany). 1× PBS, 5 mg/mL bovine serum albumin (BSA) and BCA protein assay kit were purchased from Solarbio Science and Technology Co., Ltd. (Beijing, China). Anti-TSG101 antibody, anti-CD9 antibody, anti-CD63 antibody and goat anti-rabbit IgG-HRP antibody were purchased from HuaAn Biotechnology Co., Ltd. (Hangzhou, China). Polyvinylidene fluoride (PVDF) microporous transfer membrane was purchased from Merck (Darmstadt, Germany). Hepes-Tris precast PAGE gel for Bio-Rad and 1× Western Block Buffer in TBST with Non-Fat Milk were purchased from Meilun Biotechnology Co., Ltd. (Dalian, China). All aqueous solutions were prepared using ultrapure water (18.2 MΩ cm) purified by Milli-Q system from Merck Millipore (Darmstadt, Germany).

Characterization of isolated exosomes from HC and MN patients

For morphology characterization of exosomes, the exosome eluent was dropped onto a 200-mesh formvar carbon-coated copper grid and resting for 10 min. After blotting up the remaining liquid, 2% phosphotungstic acid was dropped onto the copper grid,

incubated for 3 min to negative stain exosomes. Then the grid was dried and observed *via* JEM-2010 transmission electron microscopy (TEM) at 120 kV (JEOL, Tokyo, Japan)

The particle size and concentration of exosomes were characterized by nanoparticle tracking analysis (NTA) using ZetaView PMX 110 (Meerbusch, Germany). The device was calibrated by 110 nm polystyrene particles. The exosome eluent was diluted 1000 times by PBS in advance, temperature and pH of which maintained 23°C/7.0 during measurement. NTA measurement was recorded and analyzed at 11 positions.

The characteristic proteins in urine and exosome were identified by Western blot. First, the concentrated urine and its exosomal eluent were treated with 5 × SDS-PAGE protein loading buffer and heated at 100 °C for 5 min to denature the proteins, after calculating protein concentration using BCA protein assay kit. Next, the proteins were separated using SDS-PAGE and transferred onto the PVDF membrane through the wet membrane transfer device. The PVDF membrane was blocked for 1 h at room temperature in 1× Western Block Buffer in TBST with Non-Fat Milk, and incubated with primary antibodies at 4 °C overnight as follows: anti-TSG101 antibody, anti-CD63 and anti-CD9 antibody. After incubation with goat anti-rabbit IgG-HRP antibody for 2 h, blots were observed using ChemiDoc^{TM} Touch Imaging System (Bio-Rad Laboratories, Hercules, USA).

Supplementary figures

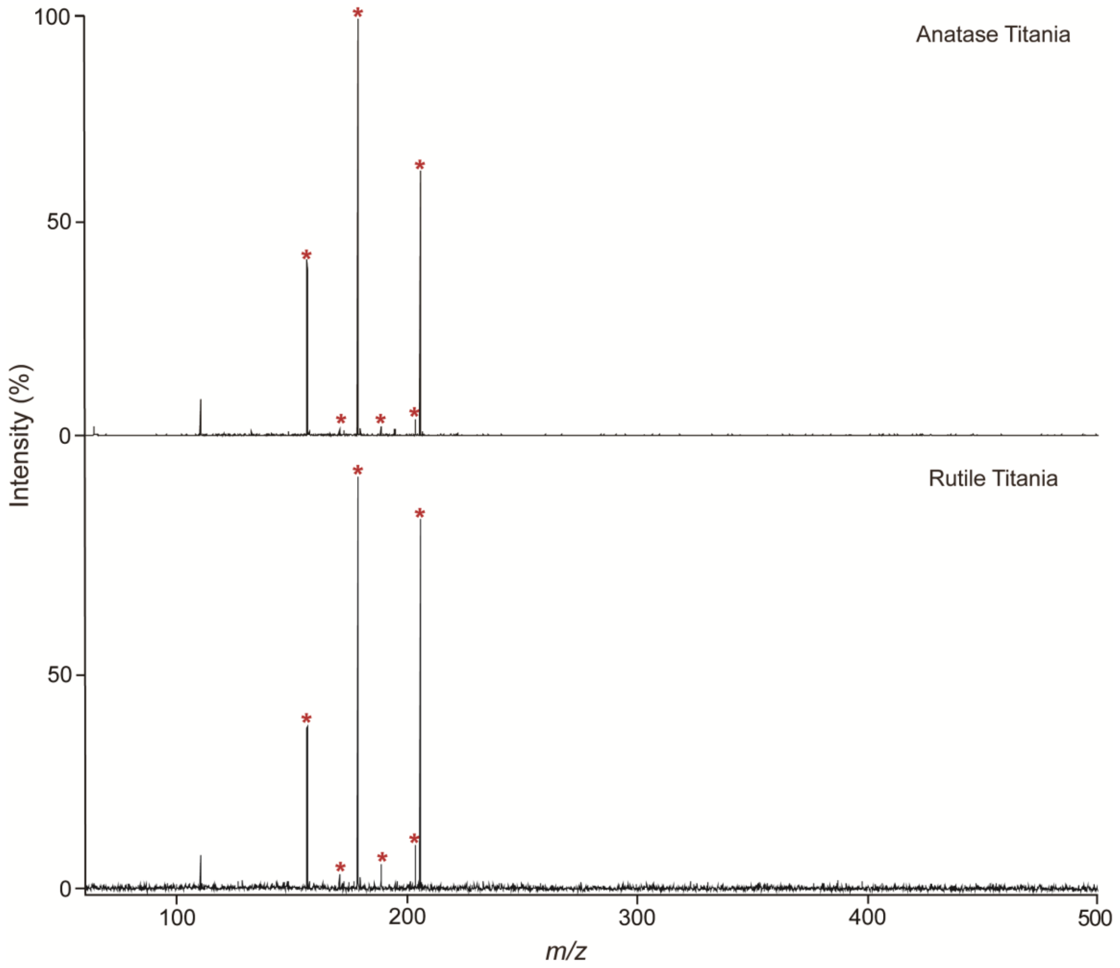

Figure S1. Mass spectra of 2 mg/mL standard metabolites mixture using anatase titania and rutile titania, peaks with signal-to-noise ratio > 3 and relative intensity > 2% from the standard metabolites were annotated with asterisk.

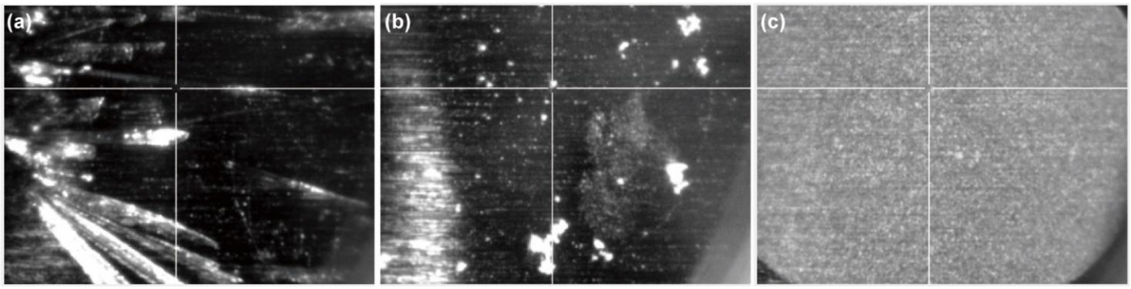

Figure S2. Images of (a) DHB-histidine cocrystal, (b) CHCA-histidine cocrystal and (c) designer titania-histidine cocrystal dispersed on the target plate. Concentration: DHB (5 mg/mL), CHCA (5 mg/mL), designer titania (1 mg/mL), and histidine (200 μmol/L).

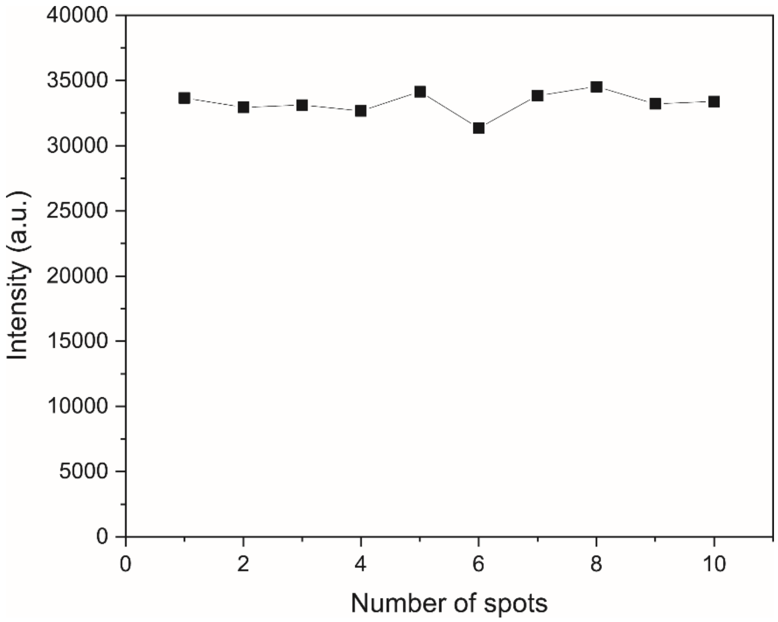

Figure S3. Mass signal intensities of histidine (200 μmol/L) repeatedly acquired from 10 sample spots using designer titania as matrix.

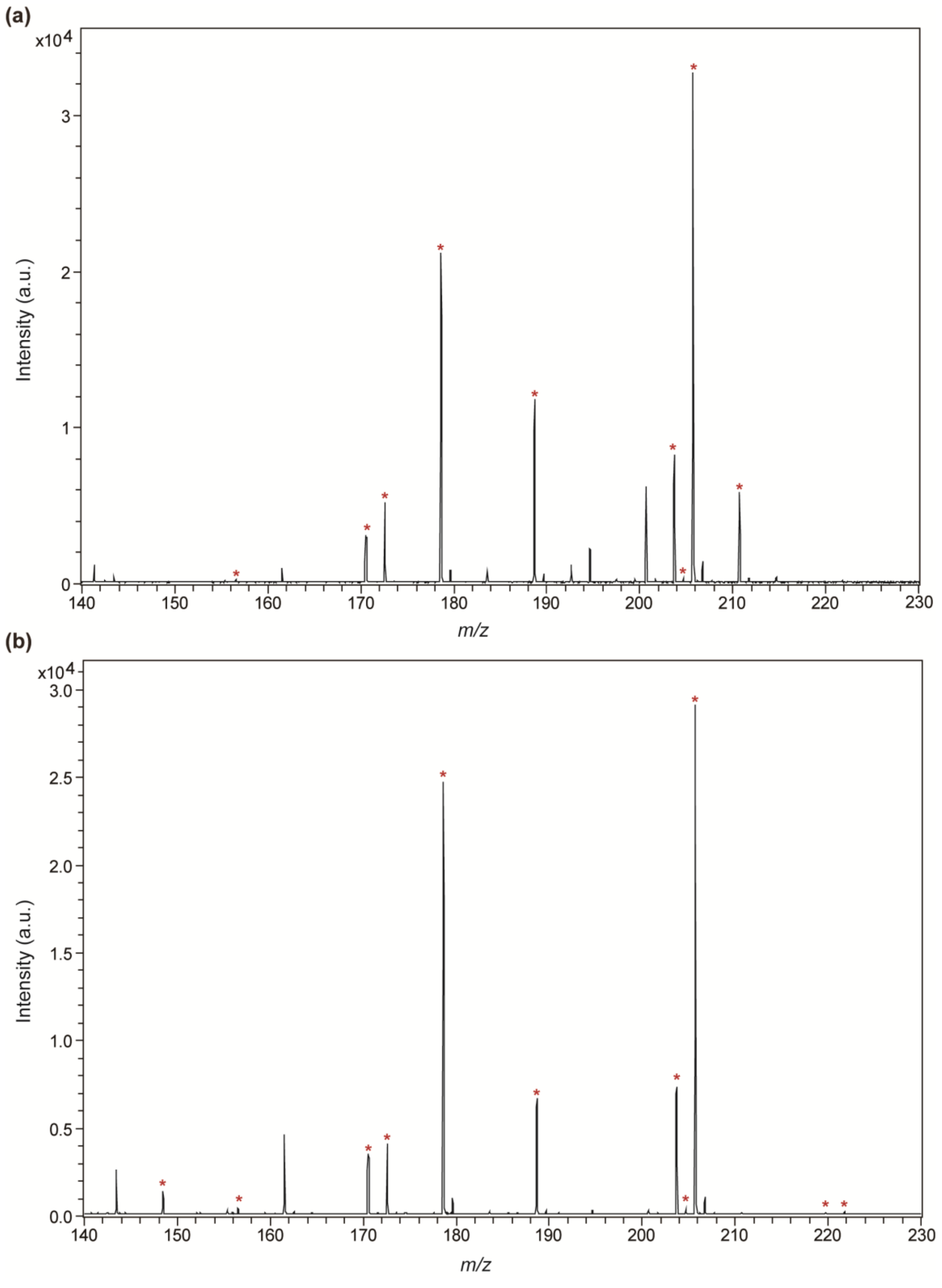

Figure S4. Mass spectra of 1 mg/mL standard metabolites mixture in 0.5 mol/L NaCl (a) and 2.5 mg/mL BSA (b) using designer titania as matrix.

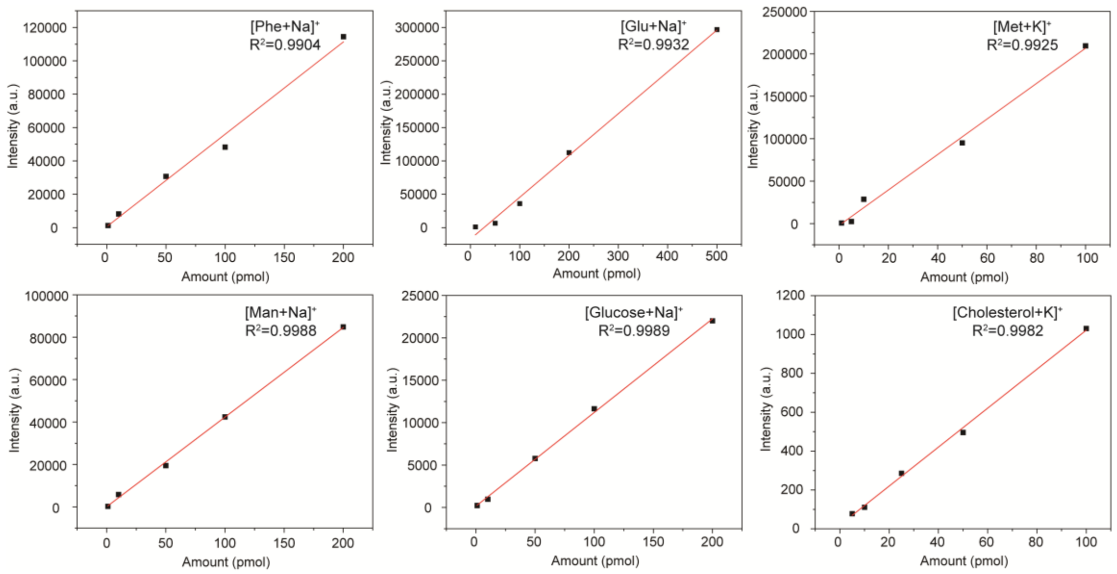

Figure S5. The calibration curve of standard metabolites obtained by designer titania-assisted LDI-MS.

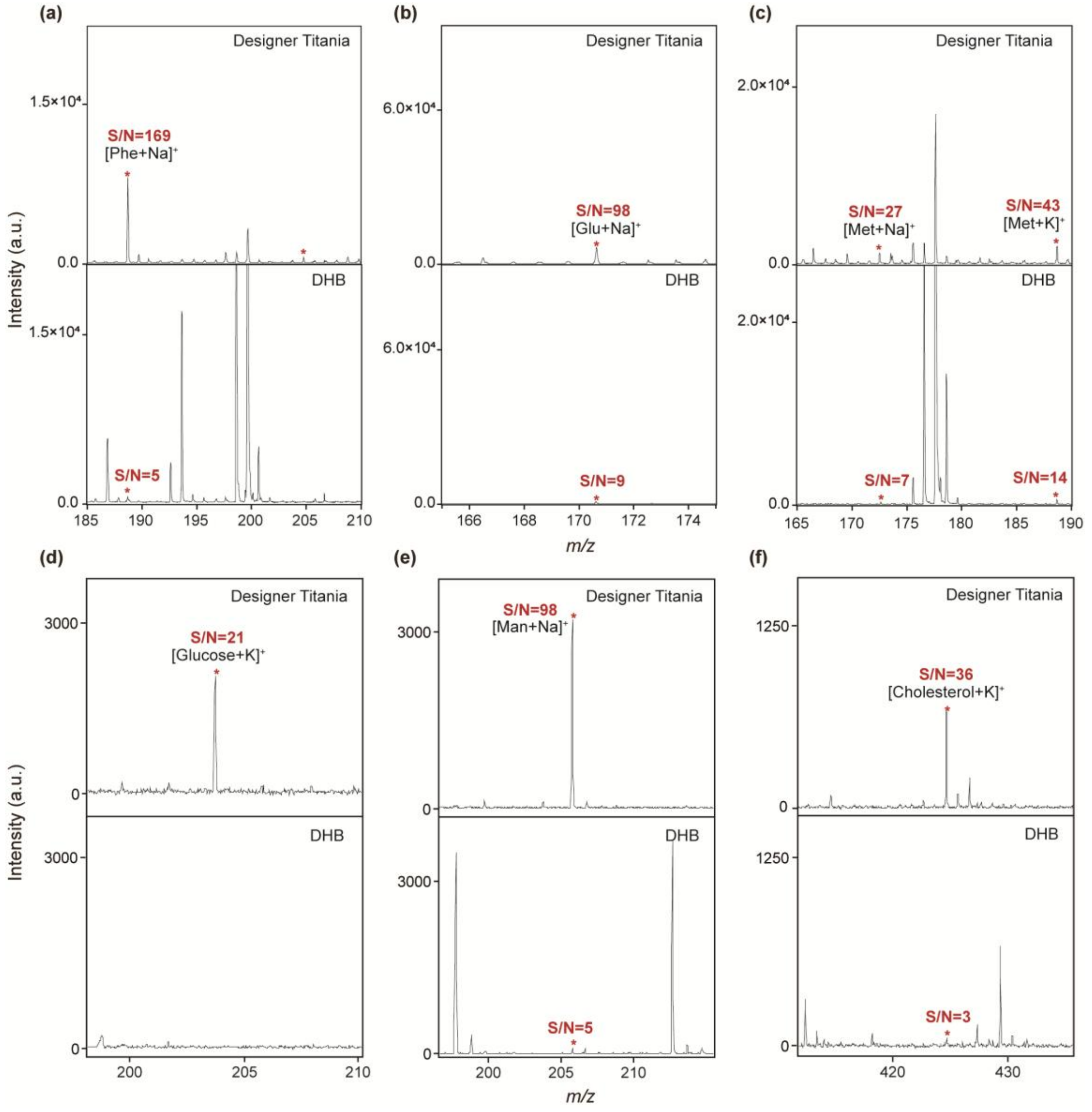

Figure S6. Mass spectra of (a) 10 pmol phenylalanine, (b) 50 pmol glutamic acid, (c) 5 pmol methionine, (d) 5 pmol mannitol, (e) 5 pmol glucose and (f) 50 pmol cholesterol using designer titania and DHB as matrix.

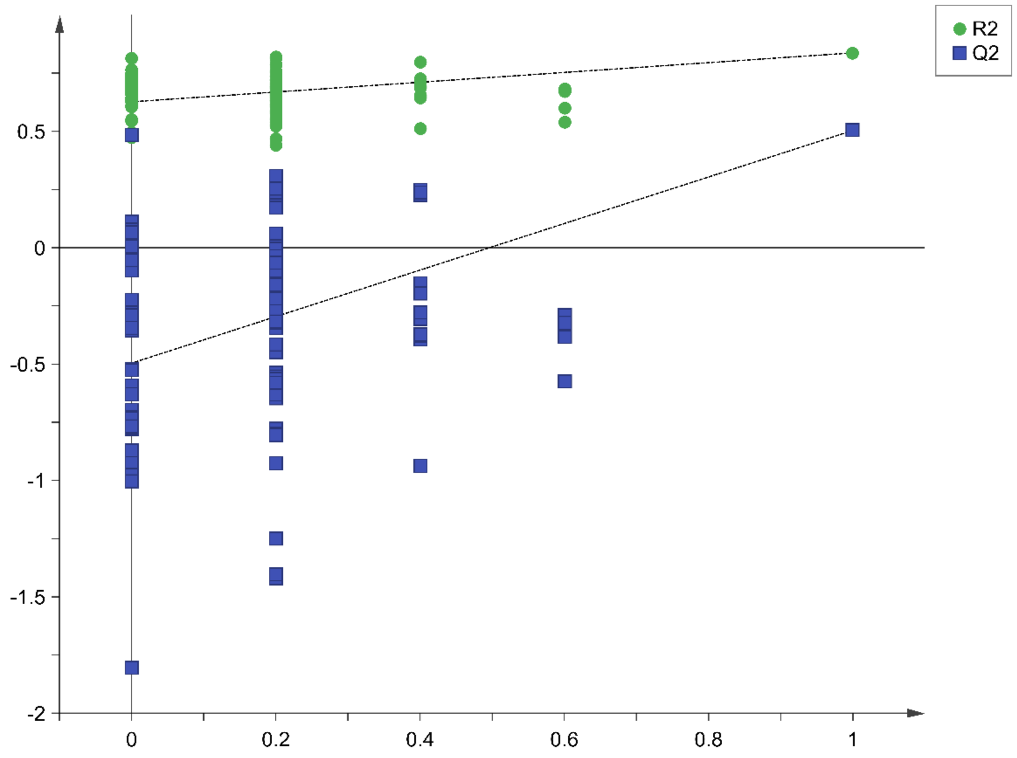

Figure S7. The permutations plot for OPLS-DA of HC vs MN patients.

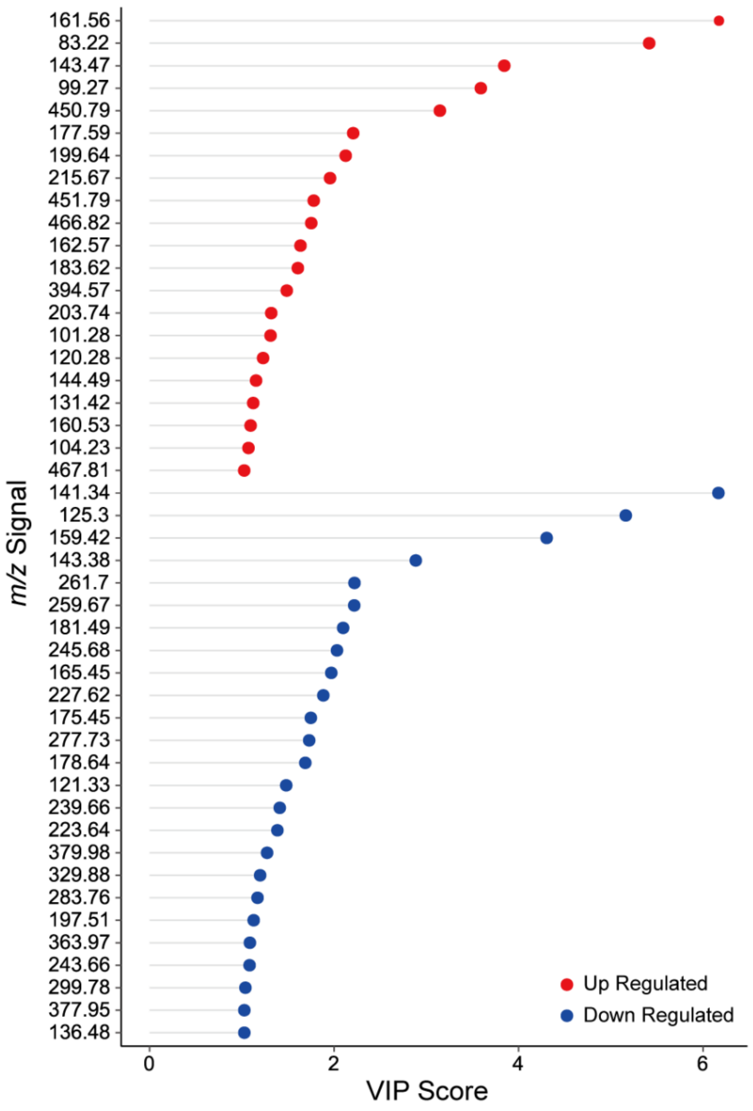

Figure S8. Dotchart of 46 *m/z* signals with VIP score more than 1 between HC and MN patients. Signals up and down regulated in MN were marked in red and blue, respectively.

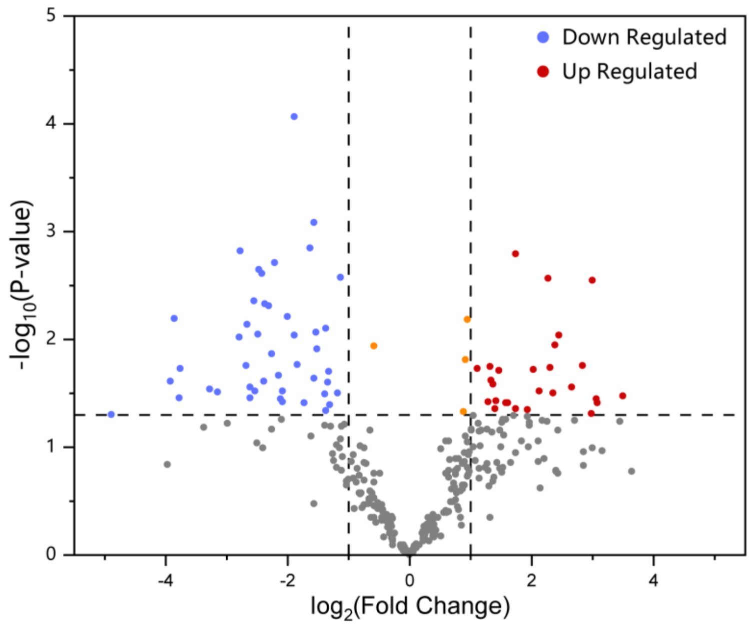

Figure S9. Volcano plot based on metabolic patterns of urinary exosomes, 72 m/z signals with p value < 0.05 and fold change >2 were colored.

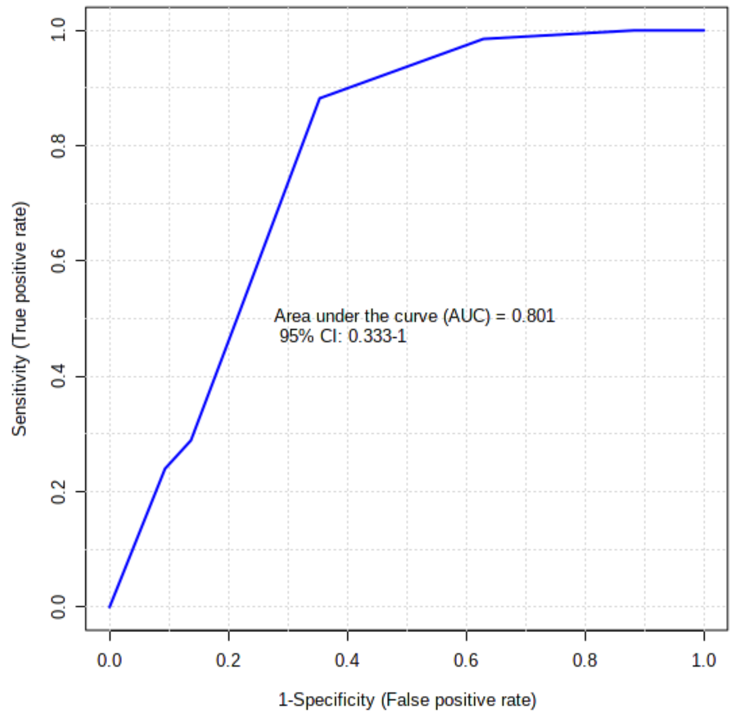

Figure S10. ROC curve based on urine metabolic patterns.

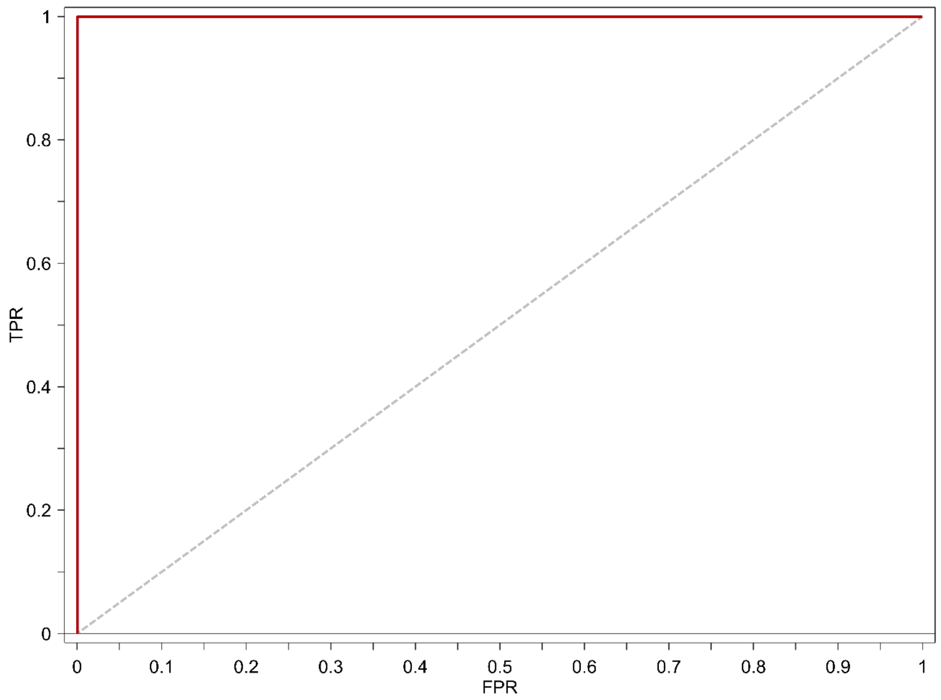

Figure S11. ROC curve of prediction set classification, based on 27 potential biomarkers and the OPLS-DA model established by the observations.

Supplementary tables

Table S1. 27 significant *m/z* signals selected from metabolic patterns of urinary exosomes.

m/z	Putative metabolite	Adduct type	VIP Score	*p* value	log_2(FC)	AUC
161.56	-	-	6.17211	0.04378	1.74481	0.765
83.22	-	-	5.41574	0.00163	1.74442	0.915
125.30	Cadaverine	$[C_5H_{14}N_2+Na]^+$	5.16193	0.00267	-1.12504	0.865
159.42	-	-	4.30430	0.00141	-1.63581	0.875
143.47	-	-	3.84462	0.00928	2.45807	0.91
143.38	-	-	2.88562	0.01722	-1.83753	0.74
261.70	Succinylaminoimidazole carboxamide	$[C_8H_9N_5O_3+K]^+$	2.22107	0.00151	-2.76690	0.895
199.64	Indole-5-carboxylic acid	$[C_9H_7NO_2+K]^+$	2.12560	0.00286	3.00252	0.865
181.49	N-Nitrosodibutylamine	$[C_8H_{18}N_2O+Na]^+$	2.09777	0.01977	-1.31552	0.77
245.68	4-(2-Aminophenyl)-2,4-dioxobutanoic acid	$[C_{10}H_9NO_4+K]^+$	2.03137	0.00649	-3.86036	0.87
165.45	Nonanal	$[C_9H_{18}O+Na]^+$	1.96919	0.00227	-2.46156	0.785
215.67	N-Formyl-L-methionine	$[C_6H_{11}NO_3S+K]^+$	1.95600	0.02767	2.65839	0.82
277.73	Dihydrobiopterin	$[C_9H_{13}N_5O_3+K]^+$	1.72974	0.03248	-1.38530	0.765
162.57	-	-	1.63562	0.03874	1.60994	0.735
183.62	-	-	1.60695	0.04886	2.98041	0.72
394.57	Arachidoyl Ethanolamide	$[C_{22}H_{45}NO_2+K]^+$	1.48690	0.03614	3.05804	0.85
239.66	Cysteine-S-sulfate	$[C_3H_7NO_5S_2+Na]^+$	1.41084	0.02442	-3.92559	0.775
223.64	Cysteine-S-sulfate	$[C_3H_7NO_5S_2+K]^+$	1.38534	0.05000	-4.88670	0.79
101.28	-	-	1.31118	0.01135	2.38761	0.91

379.98	Lactosamine	$[C_{12}H_{23}NO_{10}+K]^+$	1.27356	0.00738	-2.66328	0.855
329.88	2-Deoxy-2,3-dehydro-n-acetylneuraminic acid	$[C_{11}H_{17}NO_8+K]^+$	1.19813	0.01865	-3.75475	0.715
283.76	O-Sulfotyrosine	$[C_9H_{11}NO_6S+Na]^+$	1.17029	0.00490	-2.30936	0.79
160.53	-	-	1.09499	0.04509	1.93206	0.76
363.97	Urothion	$[C_{11}H_{11}N_5O_3S_2+K]^+$	1.08703	0.00951	-2.78438	0.79
299.78	O-Sulfotyrosine	$[C_9H_{11}NO_6S+K]^+$	1.03852	0.00623	-1.99263	0.785
377.95	6-Hydroxy-5-methoxyindole glucuronide	$[C_{15}H_{17}NO_8+K]^+$	1.02688	0.00914	-1.89320	0.795
467.81	Tetrahydrofolic acid	$[C_{19}H_{23}N_7O_6+Na]^+$	1.02543	0.03373	3.50398	0.72

Table S2. The related *m/z* signals from metabolic patterns of primitive urine.

m/z	VIP Score	*p* value	$\log_2$(FC)	AUC
83.23	2.78939	0.038	1.709	0.67
97.19	2.73542	0.001204	2.4883	0.815
152.52	2.51914	0.050705	-2.2177	0.715
450.81	2.1619	0.070131	-2.5731	0.745
112.29	1.806	0.085892	-2.69	0.585
136.50	1.60514	0.32399	0.82522	0.61
152.54	1.39879	0.35019	-0.95409	0.57
81.14	1.39077	0.11282	0.97594	0.735
113.24	1.38851	0.15814	1.2687	0.73
99.27	1.28965	0.66284	-0.31257	0.58
172.57	1.17081	0.19441	-1.8675	0.585
450.79	1.08517	0.69204	-0.62422	0.52
88.19	1.00923	0.13328	-1.0371	0.58
114.40	0.900805	0.009165	-2.0778	0.795
86.31	0.692245	0.018439	-4.8689	0.915

Table S3. Summary of 10 HC and 10 MN patients.

Subjects	Sex	Age
Healthy control	Male	36
Healthy control	Male	53
Healthy control	Male	77
Healthy control	Male	48
Healthy control	Male	29
Healthy control	Female	53
Healthy control	Female	78
Healthy control	Female	56
Healthy control	Female	69
Healthy control	Female	33
MN patient	Male	51
MN patient	Male	82
MN patient	Male	75
MN patient	Male	78
MN patient	Male	38
MN patient	Female	57
MN patient	Female	45
MN patient	Female	58
MN patient	Female	33
MN patient	Female	30
